# Association Between Perceived Stress and Alzheimer's Biomarkers in a Cohort of Middle‐Aged, High‐Risk Adults

**DOI:** 10.1002/alz70856_106513

**Published:** 2026-01-07

**Authors:** Chloe Park, Whitney Wharton, William T. Hu, Hanfeng Huang, Patrick Gavin Kehoe, James Scott Miners, Danielle D Verble, Henrik Zetterberg, Bruno L. Hammerschlag, Lynn Marie Trotti, Karima Benameur, Brittany Butts

**Affiliations:** ^1^ Emory University, Atlanta, GA, USA; ^2^ Rutgers‐Robert Wood Johnson Medical School, New Brunswick, NJ, USA; ^3^ Georgetown University School of Medicine, Washington, DC, USA; ^4^ University of Bristol, Bristol, United Kingdom; ^5^ University of Bristol, Bristol, Horfield, United Kingdom; ^6^ Department of Psychiatry and Neurochemistry, Institute of Neuroscience and Physiology, The Sahlgrenska Academy, University of Gothenburg, Mölndal, Sweden; ^7^ Athinoula A. Martinos Center for Biomedical Imaging, Massachusetts General Hospital, Harvard Medical School, Charlestown, MA, USA; ^8^ Emory University School of Medicine, Atlanta, GA, USA; ^9^ Emory University, Altanta, GA, USA

## Abstract

**Background:**

Studies suggest a chronic stress‐activated pathway in the development of Alzheimer's disease (AD) and cognitive decline, underscoring the need to better understand mechanisms of stress and downstream impacts. Inflammation and vascular dysfunction caused by subjective stress may contribute to pathology in AD. Here, we investigated how stress relates to AD biomarkers and cognition in a cohort of B/AA (Black/African Americans) and NHW (non‐Hispanic White) participants in the NIH‐funded ASCEND study.

**Method:**

Cognitively normal, middle‐aged (45‐65 years) B/AA and NHW adults with a parental history of AD were enrolled in a 2‐year observational study. CSF was collected for measurement of Aβ and tau, MMPs (MMP‐2), and markers of vascular function (sPDGFRβ, VCAM‐1) and inflammation (IL‐7). At the same visit, blood was collected for APOE genotyping and inflammatory markers (IL‐12), in addition to the Digit Span for measurement of cognition. The 10‐question Perceived Stress Scale (PSS) assessed perceived stress.

**Result:**

Participants (*N* = 50, mean age 58 ± 7 years) were mostly female (66%), well‐educated (84% college or higher), 36% earning ≥ $40K, 30% B/AA, and 48% APOE ε4 positive. PSS was positively correlated with IL‐7 (r=.392, *p* = .024), IL‐12 (r=.375, *p* = .021), MMP‐2 (r=.333, *p* = .029), VCAM‐1 (r=.352, *p* = .033), and Aβ1‐42 (r=.328, *p* = .032), and negatively correlated with the number correct on backwards Digit Span (r=‐.365, *p* = .009). Linear models showed sPDGFRβ was positively associated with MMP‐2 (r=.409, *p* = .008) and VCAM‐1 (r=.473, *p* = .005).

**Conclusion:**

Higher perceived stress was associated with higher central and systemic inflammation in a cohort of cognitively normal, middle‐aged individuals at risk for AD. Stress was associated with CSF markers of MMP‐2, linked with early AD pathology, and VCAM‐1, which is involved in vascular dysfunction. SPDGFRβ in CSF was also positively correlated with MMP‐2 and VCAM‐1, suggesting that these processes may elicit blood brain barrier breakdown. The correlation between stress and Aβ1‐42 reflects that participants were cognitively unimpaired, but higher stress related to lower digit span scores suggests that working memory and executive function are impacted. A larger sample size is required for more robust data analysis. More research is needed examining mechanisms of stress to lower AD risk, particularly in diverse populations at high risk for AD.

